# Surveillance of bacterial colonization and infection in high-risk neonates admitted to tertiary referral hospital in Indonesia

**DOI:** 10.1186/s12879-026-13724-6

**Published:** 2026-06-06

**Authors:** Riyadi Adrizain, Fadila Dyah Trie Utami, Agung Putri Mayasari Bhaskara, M. Nabil Sulthoni Eralsyah, Elis Ecin Kurniasih, Natasha Amalda Ediwan, Adhi Kristianto Sugianli, Leonardus Widyatmoko, Tetty Yuniati

**Affiliations:** 1 Department of Child Health, Faculty of Medicine, Universitas Padjadjaran / Dr. Hasan Sadikin General Hospital, Bandung, West Java Indonesia; 2 Infection and Prevention Control Nurse, Infection and Prevention Control Committee, Dr. Hasan Sadikin General Hospital Bandung, West Java, Indonesia; 3 Department of Supporting Medicine, Dr. Hasan Sadikin General Hospital, Bandung, West Java Indonesia; 4 Department of Clinical Pathology, Faculty of Medicine, Universitas Padjadjaran, Bandung, West Java Indonesia

**Keywords:** Surveillance, Infection, Neonates, Colonization, Lower-Middle Income Country

## Abstract

**Background:**

Neonatal mortality remains high in Indonesia, particularly at our center, Dr. Hasan Sadikin General Hospital, where sepsis is the most common cause of death. However, data on colonization dynamics-which are essential for preventing Multi-drug Resistance Organism (MDRO) acquisition-remain limited, particularly in lower-middle income country (LMIC). This study aims to investigate the dynamics of bacterial colonization among high-risk neonates and determine the pattern and timing of MDRO acquisition.

**Methods:**

Between May and December 2025, 46 neonates were included in this prospective cohort study.

**Results:**

MDRO colonization was detected in 32 neonates (69.6%) who were hospitalized for ≥ 48 h in patient. Outborn neonates had significantly higher risk of MDRO colonization compared with inborn neonates (RR 1.61; 95% CI 1.11–2.33; *p* = 0.018), with 93.3% outborn neonates testing positive for MDRO colonization. From 146 samples, 110 bacterial isolates were identified, 80 of these (72.7%) were MDROs, the predominant MDRO isolates were *Klebsiella pneumoniae* and *Escherichia coli*, including carbapenem-resistant and ESBL-producing strains. MDRO colonization was detected at admission and increased dynamically during hospitalization particularly among outborn neonates. Blood cultures were performed for 21 neonates with suspected infection, of these, 8 (38.1%) were positive, and 5 (62.5% of the positive cultures) yielded MDROs.

**Conclusions:**

These findings highlight the importance of routine surveillance and consistent adherence to infection prevention control measures, including standard cleaning and disinfection protocols. Regular evaluation of separation between inborn and outborn units also essential to reduce the burden of MDROs and improve neonatal outcomes.

## Background

The first 30 days of life represent the highest-risk period for child survival. The World Health Organization (WHO) reported 2.3 million neonatal deaths in 2022 [1]. Neonatal sepsis is a leading cause of mortality [2], and infections caused by Gram-negative bacteria-particularly multidrug-resistance (MDR) pathogens such as *Klebsiella pneumoniae*-have been increasing in recent years [3]. Neonatal sepsis caused by MDR pathogens is associated with greater vulnerability and poorer prognosis due to the complexity of the disease and the frequent failure of first-line antibiotic therapy [4].

Pathogen colonization plays a significant role in the development of sepsis. Multidrug-resistance organism (MDROs) may be acquired within an individual due to antibiotic selection pressure or they can be transmitted vertically (from mother to neonate) [5] or horizontally (from the healthcare environment) [6]. Risk factors for MDR infections include healthcare exposure, maternal history of infection, recent antibiotic use, the presence of invasive medical devices, and prolonged hospitalization. Notably, nosocomial infections-which frequently involve these MDR pathogens-are defined as those occurring ≥ 48 h after admission to a healthcare facility [7]. Neonates are highly vulnerable to these infections due to their immature immune system [8]. Antimicrobial resistance develops through several mechanisms, such as the transfer of resistance genes, efflux pump activity, reduced membrane permeability, the production of drug-inactivating enzymes, and structural modification of drug targets [9].

This study aimed to determine the prevalence of bacterial colonization and infection, assess the timing of MDRO acquisition among high-risk neonates, and evaluate whether colonization was present at admission or acquired during hospitalization. Additionally, the study explored potential associations between clinical variables and MDRO acquisition. Although neonatal mortality has been widely studied, the site-specific data on the rate and dynamics of bacterial colonization necessary to determine how and when optimal prevention of MDR pathogen acquisition can be implemented remain limited. Therefore, to support the government effort in reducing the number of neonatal deaths, surveillance studies on microbial colonization and MDROs are essential to understand the underlying causes and generate actionable insights.

## Materials and methods

### Study design

We conducted a prospective cohort study of neonates from delivery until hospital discharge to evaluate the incidence and risk factors of potentially pathogenic colonization.

### Study site and sampling

This study was conducted at Dr Hasan Sadikin General Hospital in Bandung, West Java, Indonesia, from 6th May to 6th December 2025. At Dr. Hasan Sadikin General Hospital in Bandung, Indonesia approximately 220 neonatal deaths are reported over the past year, with sepsis being the most common cause. The study included neonates who were delivered at the study site (Group 1) or Neonates transferred from other institution (Group 2), based on specific criteria. Inclusion criteria were live-born infants aged ≤ 28 days, neonates born to mothers ≥ 18 years of age; and high-risk admissions (defined by prematurity, small for gestational age, sepsis risk factors such as maternal fever and prolonged rupture of membranes, gestational diabetes, suspected or confirmed congenital abnormalities, fetal distress, twin pregnancy, or a clinical impression requiring high-risk admission). Neonates in Group 2 had a median age of 8 days at admission (1QR, 2–15 days). Exclusion criteria were maternal age < 18 years, stillbirth, imperforate anus, and not meeting the high-risk admission criteria.

**Fig. 1:**
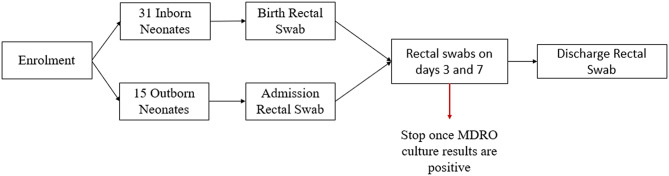
Swab time points for participants

A total of 46 neonates were included to provide preliminary data on MDRO colonization and potential associated risk factors in the neonatal unit. Given the exploratory and pilot nature of the study, as well as the limited prior data regarding MDRO colonization in neonates in our setting, the sample size was considered acceptable based on applied rule of thumb recommendation for pilot studies [10], in which approximately 30 participants are generally considered sufficient to generate preliminary estimates and inform future larger studies. Comparative analyses were therefore interpreted as exploratory and hypothesis-generating rather than confirmatory. The data collection process is illustrated in Fig. 1, including the timing of rectal swab collection in this study.

### Definition

In-born was defined as infant born within the study hospital, where the mother is admitted for delivery and the birth occurs at the same facility.

Outborn was defined as infant delivered outside the study hospital and referred or transferred to the study hospital for further care.

MDRO was defined as a microorganism non-susceptible to at least one agent in three or more antimicrobial categories.

### Data collection

Potentially pathogenic swabs for MDR pathogens were collected from neonate patients. Rectal swabs were collected using standard swabs with Amies transport medium. Samples were transported at room temperature conditions and handled as fresh specimens for prompt processing. The swab was inserted 1 cm into the anus and rotated for 10 s, for neonates delivered at the hospital, Rectal swabs were collected within 15 min of birth and assumed to represent vertically acquired colonization. Collected swabs were immediately transported to the microbiology laboratory where they were processed on the same day as collection. If samples cannot be processed the day of the collection, they will be stored in a 4 °C refrigerator for a maximum of 24 h.

Samples were inoculated onto blood agar and MacConkey agar and incubated at 37 °C in aerobic conditions for 24 h. Clinical data were obtained from patients’ medical records.

Suspected bacterial colonies were identified and tested for antimicrobial susceptibility using the BD Phoenix automated analyzer (Becton Dickinson, NJ, USA). MDRO isolates were identified based on alert notifications generated by the BD Epicentre system (Becton Dickinson, NJ, USA). Antimicrobial susceptibility testing (AST) interpretation was performed according to the Clinical and Laboratory Standards Institute (CLSI) M100 guidelines, 2025 edition. Confirmatory testing for extended-spectrum beta lactamase (ESBL) production and Carbapenem-resistant Enterobacterales (CRE) was not performed. Interpretations were done by BD Epicenter according to patterns of AST results. Automated system performance and resistant interpretation were validated weekly using American Type Culture Collection (ATCC) quality control strains in accordance with the manufacturer’s recommendations (Escherichia coli ATCC 25922). Clinical data were obtained from patients’ medical records.

### Data management and analysis

All statistical analyses were performed using MedCalc Statistical Software, version 22.023. Categorical variables were summarized as frequencies and percentages while continuous variables were described using median. Comparisons between groups were performed using The Chi-squared test or Fisher’s exact test for categorical variables, depending on expected cell counts. Continuous variables were compared using Mann-Whitney U test. Given the small sample size and exploratory nature of the study, no multivariable analysis was performed. A p-value of < 0.05 was considered statistically significant, and findings were interpreted with caution.

## Result

During the study period from May to December 2025. Among 46 neonates were included in this study, 31 inborn and 15 outborn admitted to our center. All analyses were conducted at the patient level, and MDRO status was defined based on the overall colonization status during hospitalization. The characteristics of neonates according to MDRO status are presented in Table 1. 32 (69.6%) acquired MDRO colonization, while 14 (30.4%) did not. No significant associations were observed between MDRO acquisition and gender, mode of delivery, gestational age, low birth weight, maternal comorbidity, or mortality outcome (all *p* > 0.05). Regarding respiratory support, neonates receiving invasive ventilation had a significantly higher risk of MDRO acquisition compared with those receiving room air support (RR 1.67, 95% CI 1.08–2.59; *p* = 0.022). Non-invasive ventilation was not significantly associated with MDRO acquisition. Swab collection within 48 hours was significantly associated with increased odds of the outcome compared with collection after 48 hours (RR 1.64, 95% CI 1.26–2.12; *p* = 0.020). Place of birth was significantly associated with MDRO acquisition. Outborn neonates had a higher risk of MDRO acquisition than inborn neonates (RR 1.61, 95% CI 1.11–2.33; *p* = 0.018). Antibiotic exposure was strongly associated with MDRO acquisition. Neonates exposed to antibiotics had a 2.75-fold higher risk of MDRO acquisition compared with those without antibiotic exposure (RR 2.75, 95% CI 1.50–5.02; *p* < 0.001). Duration of NICU stay was also associated with MDRO acquisition. Compared with short NICU stay (1–3 days), moderate stay (4–28 days) increased the risk of MDRO acquisition by 2.50 times (RR 2.50, 95% CI 1.19–5.27; *p* = 0.016), while prolonged stay (> 28 days) increased the risk threefold (RR 3.00, 95% CI 1.46–6.19; *p* = 0.003).

**Table 1:** Neonatal characteristics by MDRO acquisition status

Characteristics	MDRO Acquisition				*p* Value *	RR (95% CI)
	Positive		Negative			
	*n*	%	*n*	%		
**1. Gender**						
Male	18	56.3	5	35.7	0.337	1.29 (0.87–1.90)
Female	14	43.7	9	64.3		Reference
**2. Mode of Delivery**						
Caesarean Section	15	46.9	5	35.7		Reference
Vaginal Delivery	17	53.1	9	64.3	0.535	1.15 (0.79–1.67)
**3. Breathing Support**						
Room Air	10	31,3	8	57.1		Reference
Non-invasive Ventilation	9	28.1	5	35.7	0.615	1.16 (0.66–2.044)
Invasive Ventilation	13	40.6	1	7.1	0.022*	1.67 (1.08–2.59)
**4. Gestational Age**						
Preterm < 37 weeks	14	43.8	3	21.4	0.195	1.33 (0.93–1.90)
Aterm 37 weeks – 41 weeks	18	56.3	11	78.6		Reference
**5. Low Birth Weight (< 2**,**500 gram)**						
Yes	18	56.3	5	35.7	0.337	1.29 (0.87–1.90)
No	14	43.8	9	64.3		Reference
**6. Existing co-morbid of the mother**						
Yes	6	18.8	0	0.0	0.157	1.54 (1.23–1.93)
No	26	81.2	14	100.0		Reference
**7. Surgical History**						
Yes	0	0.0	0	0.0	N/A**	N/A**
No	32	100.0	14	100.0		
**8. Outcomes**						
Death	3	9.4	1	7.1	1.000	1.09 (0.60–1.98)
Alive	29	90.6	13	92.9		Reference
**9. TimeSwab Point**						
Before < 48 h	10	31.3	0	72.0	0.020*	1.64 (1.26–2.12)
After ≥ 48 h	22	68.8	14	28.0		Reference
**10 Place of Birth**						
Inborn	18	56.3	13	92.9		Reference
Outborn	14	43.8	1	7.1	0.018*	1.61 (1.11–2.33)
**11. Antibiotic Exposure**						
Yes	25	78.1	1	7.1	< 0.001*	2.75 (1.50–5.02)
No	7	21.9	13	92.9		Reference
**12. NICU Stay**						
Short Stay, 1–3 days	5	15.6	10	71.4		Reference
Moderate stay, 4–28 days	20	62.5	4	28.6	0.016*	2.50 (1.19–5.27)
Long Stay, > 28 days	7	21.9	0	0.0	0.003*	3.00 (1.46–6.19)

Note: Data are presented as frequency (n) and percentage (%). The percentages were calculated within each MDRO acquisition status group. RR = risk ratio; CI = confidence interval; MDRO = multidrug-resistance organism; NICU = neonatal intensive care unit. The reference category was used as the comparison group for RR estimation. For variables with two categories, p values were obtained using Fisher’s exact test when expected cell counts were small. For breathing support and NICU stay, RR values were estimated using log-link regression models, with room air and short NICU stay as the reference categories, respectively. *p value of < 0.05 was considered statistically significant. ** RR and p value could not be calculated because no neonates had a surgery history

Upon admission, MDRO colonization was significantly more common among outborn neonates (53.33%), eight of 15 outborn neonates (53.3%) had MDRO at admission, compared with inborn neonates (6.5%) (*p* < 0.001). Outborn neonates had an 16.88-fold higher risk of MDRO at admission compared with inborn neonates (RR 16.88; 95% CI 2.34–121.60). During hospitalization, the prevalence of MDRO colonization increased to 32 of 46 neonates (69.6%). MDRO remained more frequent among outborn neonates, occurring in 14 of 15 neonates (93.3%), compared with 18 of 31 inborn neonates (58.06%) (*p* = 0.018). Outborn neonates had a 1.61-fold higher risk of MDRO during hospitalization compared with inborn neonates (RR 1.61; 95% CI 1.11–2.33) (Table 2).

**Table 2:** Comparison of MDR pathogen status between inborn and outborn neonates

Variable	Total (*n* = 46)		Inborn (*n* = 31)		Outborn (*n* = 15)		*p*-value	RR (95% CI)
	*n*	%	*n*	%	*n*	%		
Day 1 Admission								
Non MDRO	36	78.3	29	93.5	7	46.67		Reference
MDRO	10	21.7	2	6.5	8	53.33	< 0.001*	16.88 (2.34–121.60)
**During Hospitalization (≥ 48 h)**								
Non MDRO	14	30.4	13	41.94	1	0.07		Reference
MDRO	32	69.6	18	58.06	14	93.3	0.018*	1.61 (1.11–2.33)

Note: Data are presented as frequency (n) and percentage (%). MDR = multidrug-resistance; MDRO = multidrug-resistance organism; RR = risk ratio; CI = confidence interval. Inborn neonates were used as the reference group for RR estimation. The p-value was obtained using Fisher’s exact test. *p < 0.05 was considered statistically significant

**Fig. 2:**
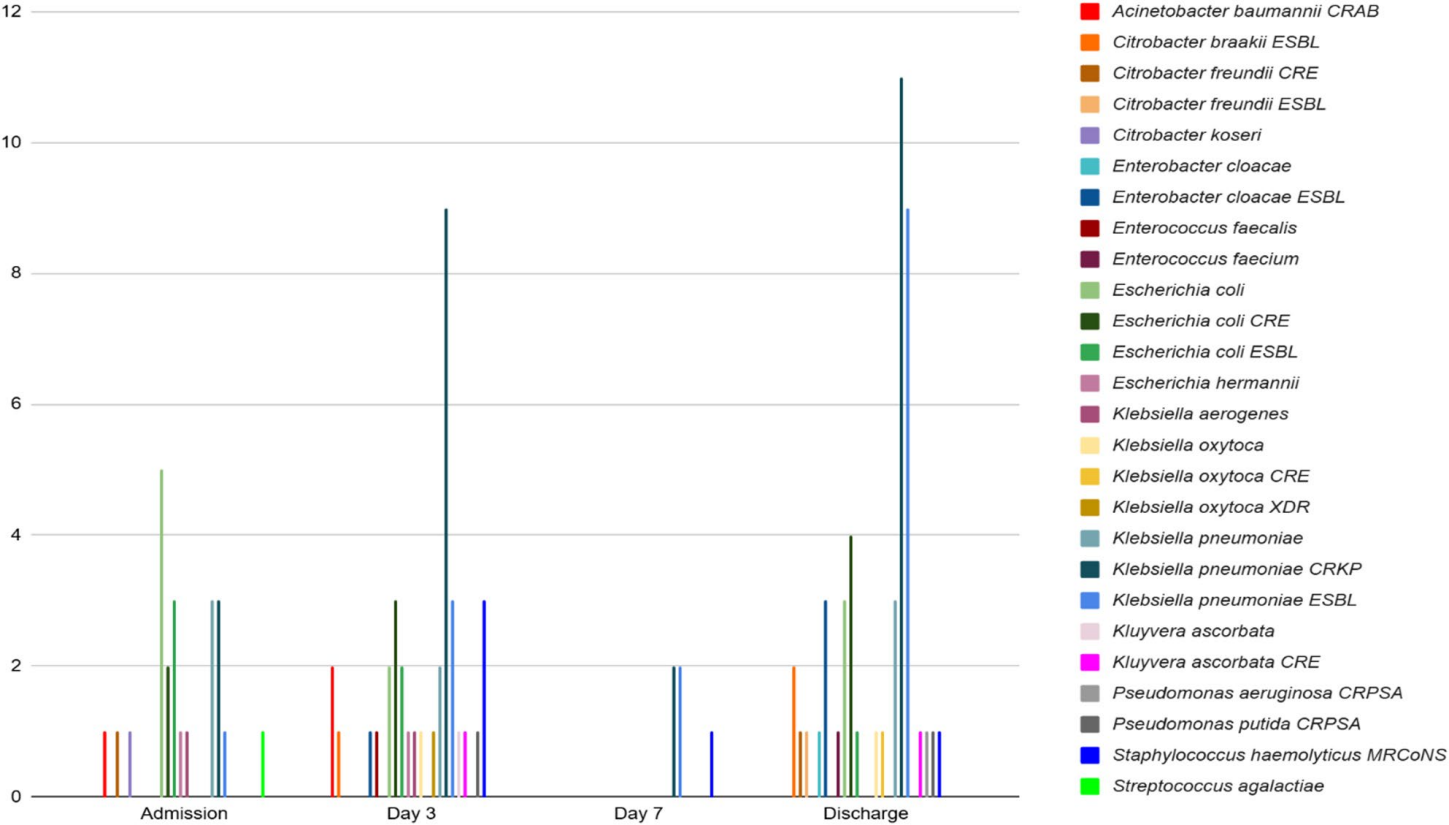
Distribution of bacterial isolates across serial rectal swab collections

In total, 146 rectal swab isolates collected from 46 neonates in Fig. 2, including repeated samples during hospitalization at birth, day 3, day 7, and discharge. Among 146 isolates, 36 yielded no bacterial growth. From the remaining positive samples, 110 bacterial isolates were identified, consisting of 102 Gram-negative and 8 Gram-positive. Overall, 80 (72.7%) isolates were classified MDROs, including 75 Gram-negative and 5 Gram-positive isolates. Serial rectal swab surveillance demonstrated that MDRO colonization was already present at the time of admission and evolved dynamically during hospitalization. At admission, colonization was predominantly caused by *Escherichia coli* (*n* = 5), carbapenem-resistant *Klebsiella pneumoniae* (CRKP) (*n* = 3), *Klebsiella pneumoniae* (*n* = 3), and ESBL-producing *Escherichia coli* (*n* = 3). Several carbapenem-resistant organisms, including *Escherichia coli* CRE and *Acinetobacter baumannii* CRAB, were also identified at baseline. By day 3 of hospitalization, the number and diversity of MDRO isolates increased substantially. CRKP became the predominant organism (*n* = 9), followed by ESBL-producing *K. pneumoniae* (*n* = 3), *E. coli* CRE (*n* = 3), and MRCoNS (*n* = 3). Additional resistant Enterobacterales species, including ESBL-producing *Enterobacter cloacae*, *Citrobacter braakii*, and carbapenem-resistant *Kluyvera ascorbata*, also emerged during this period. The number of isolates detected on day 7 was substantially lower. However, this result should be considered in the context of the study protocol, as repeat rectal swabs were not routinely collected from neonates who had already tested positive on previous sampling. These patients were subsequently re-swabbed at discharge to evaluate persistent colonization status. The colonization density decreased markedly, with only a limited number of persistent isolates remaining detectable, predominantly CRKP (*n* = 2) and ESBL-producing *K. pneumoniae* (*n* = 2). At discharge, MDRO colonization increased again, with CRKP remaining the most frequent isolate (*n* = 11), followed by ESBL-producing *K. pneumoniae* (*n* = 9), *E. coli* CRE (*n* = 4), and *E. coli* (*n* = 3). These findings suggest that MDRO colonization may begin prior to or at admission in some neonates, while additional acquisition and persistence of resistance organisms occur during NICU hospitalization.

Among 37 neonates with positive rectal swab colonization, 27 were classified as having infection-causing isolates, while 10 were considered colonizing isolates without clinical evidence of infection. Gram-negative organisms predominated in both blood and rectal swab cultures, particularly *Klebsiella pneumoniae* CRKP, *Klebsiella pneumoniae* ESBL, and *Escherichia coli* CRE/ESBL. Among 46 neonates, blood cultures were obtained from 21 due to suspected signs of infection. Eight of these 21 (40%) cultures were positive. Five of the eight positive cultures (62.5%) yielded MDROs. Of these five MDRO-positive cultures, four (80%) grew the same organism that had been previously identified as a colonizing isolate. Two of these neonates died, and all five experienced prolonged hospitalization, suggesting a high pathogen burden (Table 3).

**Table 3:** Comparison of blood culture and rectal swab culture profiles between colonizing and infection-causing isolates

Infection-causing Isolates		
NO	Rectal Swab Culture	Blood Culture
1	*Kebsiella pneumoniae* ESBL, *Klebsiella pneumoniae* CRKP, *Escherichia coli* CRE	*Klebsiella pneumoniae* CRKP
2	*Klebsiella pneumoniae* ESBL, *Klebsiella pneumoniae* CRKP	*Klebsiella pneumoniae* ESBL
3	*Escherichia coli* CRE, *Escherichia coli* ESBL, Acinetobacter baumannii CRAB, Citrobacter koseri	*Escherichia coli* ESBL
4	*Escherichia coli* ESBL, *Escherichia coli* CRE	*Pseudomonas aeruginosa*
5	*Staphylococcus haemolyticus* MRConS, *Klebsiella pneumoniae* CRKP, Citrobacter braakii ESBL	*Burkholderia cepacia*
6	*Klebsiella pneumoniae* CRKP, *Escherichia coli* CRE	*Serratia liquefaciens*
7	*Klebsiella pneumoniae* CRKP, Pseudomonas aeruginosa CRPSA	*Klebsiella pneumoniae* CRKP
8	*Klebsiella pneumoniae* CRKP, Citrobacter braakii ESBL	*Staphylococcus epidermidis* MRCONS, Acinetobacter baumannii CRAB
9	*Staphylococcus haemolyticus MRCoNS*, Klebsiella oxytoca CRE, Citrobacter freundii CRE	Negative
10	*Streptococcus agalactiae*, *Klebsiella pneumoniae* CRKP, *Klebsiella pneumoniae* ESBL	Negative
11	*Escherichia coli* CRE, *Klebsiella pneumoniae* CRKP	Negative
12	*Kluyvera ascorbate*. *Klebsiella pneumoniae* ESBL, *Klebsiella pneumoniae* CRKP	Negative
13	*Escherichia hermannii*, *Klebsiella pneumoniae*	Negative
14	*Escherichia coli* ESBL, *Klebsiella pneumoniae* ESBL, Enterobacter cloacae ESBL	Negative
15	*Escherichia coli*, *Klebsiella oxytoca* XDR	Negative
16	*Klebsiella pneumoniae* ESBL, *Escherichia coli* ESBL, *Klebsiella pneumoniae* CRKP, *Escherichia coli*	Negative
17	*Citrobacter freundii* CRE, *Klebsiella pneumoniae* CRKP, *Klebsiella pneumoniae* ESBL	Negative
18	*Escherichia coli* CRE, *Klebsiella pneumoniae* CRKP, Citrobacter freundii ESBL	Negative
19	*Klebsiella pneumoniae* ESBL	Negative
20	*Klebsiella pneumoniae*, *Klebsiella pneumoniae* CRKP, *Klebsiella pneumoniae* ESBL, *Escherichia coli*	Negative
21	*Klebsiella pneumoniae* CRKP, *Klebsiella pneumoniae* ESBL, Enterobacter cloacae ESBL	Not Performed
22	*Escherichia coli*, *Escherichia coli* ESBL	Not Performed
23	*Escherichia coli* CRE, *Klebsiella pneumoniae* ESBL	Not Performed
24	*Klebsiella pneumoniae* CRKP, *Pseudomonas putida* CRPSA	Not Performed
25	*Klebsiella pneumoniae* CRKP	Not Performed
26	*Klebsiella pneumoniae* CRKP	Not Performed
27	*Klebsiella pneumoniae* ESBL	Not Performed
**Colonizing Isolates**		
28	*Klebsiella pneumoniae* CRKP, *Enterococcus faecium*	Not Performed
29	*Escherichia coli*	Not Performed
30	*Klebsiella aerogenes*, *Klebsiella oxytoca*	Not Performed
31	*Staphylococcus haemolyticus* MRCoNS, *Enterococcus faecalis*	Not Performed
32	*Klebsiella pneumoniae*	Not Performed
33	*Kluyvera ascorbata* CRE, *Staphylococcus haemolyticus* MrConS, *Kluyvera ascorbata* CRE	Not Performed
34	*Klebsiella pneumoniae*, *Escherichia coli*	Not Performed
35	*Enterobacter cloacae* ESBL	Not Performed
36	*Acinetobacter baumannii* CRAB, *Klebsiella pneumoniae*	Not Performed
37	*Klebsiella aerogenes*, *Enterobacter cloacae*	Not Performed

*Infection-causing isolates were defined as isolates obtained from neonates diagnosed with infection who received empirical antibiotics according to clinical judgment or definitive antibiotics based on microbiological culture findings

## Discussion

The neonatal period carries the highest risk of mortality, with sepsis remaining a leading cause of death, particularly in settings with a high burden of multidrug-resistance pathogens. In this study, we found a high prevalence of MDRO colonization, with marked differences based on the timing of acquisition and place of birth, suggesting both prior healthcare exposure and hospital-acquired transmission. At admission, MDRO colonization was significantly more common among outborn neonates at compared to inborn neonates. This finding suggests that referral-related factors, including prior hospitalization, antibiotic exposure, or delivery at facilities with differing infection control practices, may contribute to early MDRO colonization. Similar findings have been reported in previous neonatal studies [11, 12], in which transferred neonates were at a higher risk of colonization with resistance organisms due to cumulative healthcare exposure. Importantly, MDRO colonization increased significantly after 48 h of hospitalization in both inborn and outborn neonates. The significantly higher proportion of MDRO-positive cases identified after 48 h compared to admission results suggests the possibility hospital-acquired colonization and possible epidemiological linkage. This aligns with other studies that have shown the risk of MDRO gut colonization increases with the duration of hospitalization [13, 14]. Although this study was not designed to evaluate infection prevention and control (IPC) practices directly, the observed association between MDRO acquisition and antibiotic exposure, prolong hospitalization, and invasive respiratory support the importance of maintaining effective IPC strategies and antimicrobial stewardship programs (ASP) in neonatal care settings including hand hygiene compliance, environmental cleaning, surveillance cultures, appropriate isolation precaution, and consideration of environmental reservoirs such as contaminated sinks and water sources. Recent studies on waterless ICU care have also highlighted the potential role of reducing the risk of contamination [15].

We also observed a significant association between MDRO colonization and prolonged hospitalization. An extended length of stay may increase exposure to resistant organisms, a finding consistent with other studies [16, 17]. Conversely, MDRO colonization itself may contribute to more complicated clinical courses [13, 18], suggesting a possible bidirectional relationship between colonization and disease progression. Invasive ventilation was significantly associated with MDRO acquisition, and these findings supported by other study [19]. Neonates requiring invasive respiratory support had a 1.67-fold higher risk of MDRO acquisition compared with those receiving room air support. This may be due to frequent handling, invasive airway procedure, prolong device use, and greater exposure to antibiotic and prolonged NICU stay among critically ill neonates, which can facilitate transmission. Antibiotic exposure was also strongly associated with MDRO acquisition, and this is align with findings from other study. Neonates exposed to antibiotic had nearly three times higher risk of acquiring MDROs compared with those without antibiotic exposure [20]. Antibiotic use may promote the selection and persistence of resistant organisms by suppressing susceptible bacterial flora.

Gram-negative organisms predominated among isolates, consistent with findings from previous studies, with *Klebsiella pneumoniae* and *Escherichia coli* accounting for the majority of MDROs [21, 22]. The high proportion of carbapenem-resistant *Klebsiella pneumoniae* (CRKP) and ESBL-producing organisms is of clinical concerning, as these pathogens are associated with limited treatment options [23] and poor outcomes [24] in neonates. The predominance of Gram-negative MDROs observed in this study aligns with regional and global trends in neonatal intensive care units, especially in low- and middle-income settings.

Among neonates evaluated for sepsis, more than one-third had positive blood cultures, and MDROs accounted for most of these infections. Notably, in 80% of MDRO-positive bloodstream infections, the causative organism matched a previously identified colonizing isolate from rectal swabs, However, this observation was based on very small number of MDRO bloodstream infection, molecular typing was also not performed, therefore, a clonal relationship between colonizing and infecting isolates cannot be confirmed. These findings may suggest a potential relationship between prior colonization and subsequent infection, but should be interpreted with caution. This is in line with previous studies that reported rectal surveillance cultures may identify MDR strains with high sensitivity and specificity [25], and colonization profiles may help guide empirical antibiotic therapy [26].

These findings support the importance of effective environmental cleaning and disinfection practices as part of infection prevention strategies, in line with existing literature. Given that the emergency department may represent an initial point of contact with a potentially higher risk of MDRO exposure, early implementation of infection control measure in this setting may be beneficial. Previous studies have demonstrated that disinfectants such as alcohol-based agents and chlorine-based agents [27] can reduce MDRO contamination on environmental surface. In addition, the use of sporicidal agents including sodium hypochlorite, hydrogen peroxide and peracetic acid [28] and chlorhexidine bathing has been associated with reduce MDRO acquisition in healthcare settings [29]. However, the implementation of these strategies should be adapted to local resources and requires further evaluation.

Overall, our findings highlight the dynamic nature of MDRO colonization in neonates, the significant contribution of hospital exposure to MDRO acquisition, and the clinical consequences of progression from colonization to infection. Early surveillance through rectal swab screening, particularly for outborn neonates and during prolonged hospitalization, may help identify high-risk patients and inform targeted infection control strategies. From an infection control perspective, strategies such as patient grouping and enhanced precautions between inborn and outborn areas may be considered to reduce cross-transmission, particularly in high-risk settings such as emergency department. When full separation is not feasible, adherence to standard precautions including changing cloths, maintaining personal hygiene, and ensuring proper use of personal protective equipment (PPE) when moving between the two sections and could be the part of protocols.

However, several limitations should be acknowledged. First, this study was conducted with a relatively small sample size and within a single center, which may limit the generalizability of the findings to other settings. Second, molecular typing was not performed, therefore, clonal relationships between colonizing and infecting isolates could not be confirmed, and transmission pathways cannot be definitively established. Third, colonization was assessed only through rectal swabs, and potential colonization at other sites, such as the skin or respiratory tract, was not evaluated, which may underestimate the overall colonization burden. In addition, the analytical approach was limited, and multivariate analysis was not performed; therefore, potential confounding factors could not be fully controlled, and the observed associations should be interpreted as exploratory. 

As a pilot study, these findings provide preliminary insights into MDRO colonization patterns and their potential clinical implications in neonates. Further studies involving larger, multicenter cohorts and incorporating molecular epidemiological methods are needed to validate these findings and better understand transmission dynamics.

## Conclusion

MDRO colonization was common at our center, particularly among outborn neonates with longer hospital stays. The observed consistency between colonizing and bloodstream isolates suggests potential clinical relevance, although causality cannot be confirm without molecular analysis. These findings support the importance of infection prevention and control measures and antimicrobial stewardship, in line with existing literature. Strategies such as optimization of cleaning and disinfection protocols including the appropriate use of disinfectants and reagents targeting specific MDROs, also separation of facilities and staff between inborn and outborn units could be considered to minimize cross-transmission of MDRO.

## Data Availability

All data generated of analysed during this study are included in this published article.

## Abbreviations

ASP: Antimicrobial Stewardship Program
CRAB: Carbapenem-Resistant *Acinetobacter baumannii*
CRE: Carbapenem-Resistant Enterobacteriaceae
CRKP: Carbapenem-Resistant *Klebsiella pneumoniae*
CRPSA: Carbapenem-Resistant *Pseudomonas aeruginosa*
ESBL: Extended-Spectrum Beta-Lactamase
IPC: Infection Prevention and Control
IQR: Interquartile Range
LMIC: Lower-Middle Income Country
MDR: Multidrug Resistance
MDRO: Multidrug-Resistance Organism
MRCONS: Methicillin-Resistant Coagulase-Negative *Staphylococci*
NICU: Neonatal Intensive Care Unit
PPE: Personal Protective Equipment
WHO: World Health Organization
XDR: Extensively Drug Resistance
